# Senescence is delayed when ramie (*Boehmeria nivea* L.) is transformed with the isopentyl transferase (*ipt*) gene under control of the SAG12 promoter

**DOI:** 10.1002/2211-5463.12191

**Published:** 2017-03-31

**Authors:** Xia An, Jingyu Zhang, Yiwen Liao, Lijun Liu, Dingxiang Peng, Bo Wang

**Affiliations:** ^1^ Key Laboratory of Crop Ecophysiology and Farming Systems in the Middle Reaches of the Yangtze RiverMinistry of Agriculture, College of Plant Science and TechnologyHuazhong Agricultural UniversityWuhanChina; ^2^ Flower Research and Development CentreZhejiang Academy of Agricultural SciencesHangzhouChina

**Keywords:** ethylene, *ipt*, leaf senescence, ramie

## Abstract

Ramie is an economically important industrial fiber crop widely planted in China, India, and other Southeast Asian and Pacific Rim countries. It plays an important role in China's economy, where ramie farming, industry, and trade provide livelihood support to about five million people. However, poor fiber production resulting from leaf senescence and leaf abscission is a significant problem. In this study, we report the successful production of transgenic ramie plants which delayed leaf senescence and enhanced biomass. Transgenic ramie plants were obtained via transformation with the *Agrobacterium tumefaciens* strain harboring the binary vector pSG529 containing the isopentyl transferase (*ipt*) gene under control of the SAG12 promoter (P_SAG__12_‐*ipt* construct). *Agrobacterium tumefaciens* strain EHA105 was used for the midrib explant transformation. The transformation frequency was 28.29%. Southern blot confirmed the integration of 1–4 copies of the *NPTII* gene into the ramie genome in the tested lines. At the fiber maturation stage, the transgenic plants had higher photosynthesis rates, chlorophyll content (SPAD values), and stronger resistance to exogenous ethylene compared with wild‐type plants.

**Abbreviations**
Cks: cytokinins
IPT: isopentenyl transferase
PCD: programmed cell death
SAG: senescence‐associated gene
WT: wild‐type

Ramie (*Boehmeria nivea* L. Gaud) of the Urticaceae 1 is a perennial bast fiber crop that originated in China 2. Therefore, it is known as ‘China grass’ in many western countries 1. Ramie is grown on about 80 000 ha, with annual fiber production of 150 000t in 2012 (FAOSTAT, http://faostat3.fao.org). It has vigorous vegetative growth and can be harvested three times a year in the Yangtze River Basin 1 and up to six times a year in well‐watered environments in the Philippines, which means that the ramie vegetative fiber yield is very high 3.

Poor fiber production resulting from leaf senescence (Fig. 1A) and leaf abscission (Fig. 1B) at the fiber maturation stage is a significant problem. Senescence is an orderly loss of normal cell functions under the control of the nucleus 4. Leaf senescence is an important feature of the later stage of development and the age‐dependent deterioration process, and leads to death 5. Leaf senescence is illustrated clearly by dramatic color changes 6. Green leaves on perennial plants including ramie turn yellow and orange before they eventually brown, die, and are discarded from the plant 6. Leaves are specialized photosynthetic organs and the plant invests considerable energy and nutrients in leaf production 7. During leaf senescence, the earliest and most drastic change in plant cellular structures is the breakdown of chloroplasts, which contain the photosynthetic machinery of the cell and carry out major biosynthesis 8. Leaf senescence is characterized by a decline in chlorophyll content 9. Chlorophyll proteins and nucleic acids are degraded during senescence, resulting in a sharp decrease in leaf photosynthetic activity 10. Leaf senescence can therefore substantially limit crop biomass accumulation. This complex process involves a sequence of changes in cellular physiology, biochemistry, and gene expression 11. Senescence is a gradual process and therefore difficult to quantify 9. Furthermore, leaf senescence can be induced by various internal and external environmental factors, such as light and a variety of plant hormones 5, 12, 13. Many studies on senescence have been carried out to better understand the leaf senescence processes in tobacco 14, *Arabidopsis* 11, 15, cotton 16, wheat 17, rice 18, and petunia 19. Several investigations have demonstrated that leaf senescence can be induced or suppressed by various phytohormones 20. Methyl jasmonate, salicylic acid, ethylene, abscisic acid, low nutrient supply, low light conditions, stress, and pathogen infection are all thought to trigger or enhance senescence, whereas cytokinins (Cks), gibberellins, and auxins have been reported to delay senescence 9, 11, 21, 22. Cks are the strongest senescence‐delaying hormones 20, 21. Increased Ck production can delay leaf senescence, whereas reduced endogenous Ck levels can result in premature senescence 21.

**Figure 1:**
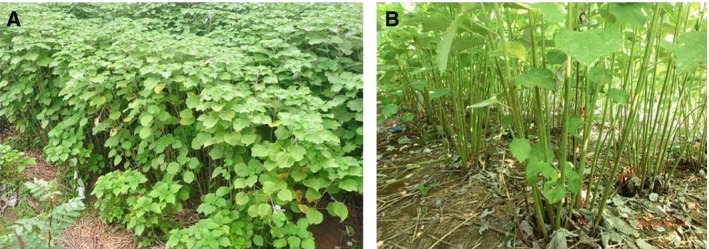
Poor production resulting from leaf senescence (A) and leaf abscission (B) is a significant problem.

The enzyme that catalyzes the rate‐limiting step of the Ck biosynthesis pathway 23 in *Agrobacterium tumefaciens* is isopentenyltransferase (IPT) 24. In an earlier study, leaf Ck concentrations were elevated and leaf senescence was delayed in transgenic plants because of overexpression of the *ipt* gene, but the high Ck levels were largely detrimental to growth and fertility 23. When the *ipt* gene was expressed under the control of a senescence‐inducible promoter (the SAG12 promoter), elevated Ck levels were localized within senescing tissues or organs and senescence was delayed without the induction of additional phenotypes associated with systemically high hormone levels 25. This approach was later successfully applied to delay the senescence process in tobacco 26, tomato 27, lettuce 23, broccoli 28, and petunia flowers 24. In addition to retardation of leaf senescence, some of these transgenic plants delayed flowering 23, 24, increased resistance to ethylene 24, 29 and drought 30, 31, and enhanced root growth 30, 32.

Previously, we developed an efficient regeneration and transformation protocol for ramie cultivar improvement by *Agrobacterium*‐mediated gene transformation using leaf midribs as explants 2. In the present study, we transformed the P_SAG12_‐*ipt* gene into the ramie cultivar ‘Huazhu No. 5’, which resulted in delayed leaf senescence and increased biomass compared with the wild‐type.

## Materials and methods

### Plant materials

‘Huazhu No. 5’ was used as the plant material in this study. It was prepared as previously described 2.

### *Agrobacterium*‐mediated transformation

The *A. tumefaciens* strain EHA105, harboring the binary plasmid pSG529, was donated by Professor Xianlong Zhang (Huazhong Agricultural University, China). *Agrobacterium*‐mediated transformation was carried out as previously described 2.

### PCR and Southern blot analysis

Genomic DNA was prepared as previously described 2. The primers for the *NPTII* gene were 5′‐TGCGAATCGGGAGCGGCGATACCG‐3′ (forward) and 5′‐TGGGCAGCACAACAGACAATCGGCTGC‐3′ (reverse) and for *ipt* were 5′‐TCGGCTTATGACTGGGCACAACAG A‐3′ (forward) and 5′‐AAGAAGGCGATAGAAGGCGATGCG‐3′ (reverse). The polymerase chain reaction (PCR) reactions of *NPTII* gene were performed as previously described 2. The PCR reactions of *ipt* gene were performed under the following conditions: 3 min at 95 °C, followed by 35 cycles of 1 min at 95 °C, 45 s at 60 °C, and 1 min at 72 °C, with a 10‐min final extension at 72 °C. Southern blot was conducted as previously described 2.

### Reverse transcriptase‐PCR analyses

Reverse transcriptase‐mediated PCR (RT‐PCR) was conducted as previously described 2 and performed using *ipt* gene‐specific primers (forward 5′‐TTGTCACTGAAGCGGGAAGG‐3′ and reverse 5′‐GATGTTTCGCTTGGTGGTCG‐3′). For qRT‐PCR, total RNA was extracted from 8‐week‐old leaves and then reverse transcribed into cDNA. The ramie *GAPDH* gene was selected as an internal control and coamplified with the primers *GAPDH* forward (5′‐TGGAAGAATCGGTAGGTTGG‐3′) and reverse (5′‐GACGCCAAAAACAGTGACAG‐3′) 33.

### Leaf senescence experiment

Transgenic and WT plants (nontransgenic ramie) grown in normal conditions were sampled at the fiber maturation stage. The 5th–8th uppermost leaves (8‐week‐old plants) were harvested and subjected to 1200 ppm ethephon. Plants were placed at 28 ± 2 °C/23 ± 2 °C (day/night) under a 16/8‐h (light/dark) photoperiod in sealed dishes. The phenotypes of these plants were examined and photographed.

### Measurements of plant height, stem diameter, relative chlorophyll content, and leaf gas exchange

The stem diameter and height of randomly selected transgenic and WT plants were measured at the beginning (control) and end (treatment) of the fiber maturation stage. Six measurements were performed for chlorophyll content using a SPAD‐502 plus (Konica Minolta, Tokyo, Japan) on corresponding functional leaves, and the average was taken. The photosynthesis rate was determined as previously described 33.

### Measurement of biomass traits

The biomass traits of randomly selected transgenic and WT plants were measured at the beginning (control) and end (treatment) of the fiber maturation stage. The biomass traits included fresh shoot weight, fresh stem weight, fresh bast weight, and dry bast weight.

### Data collection and statistical analysis

The biomass traits of randomly selected transgenic (lines 19 and 20) and WT plants were measured at the beginning (control) and end (treatment) of the fiber maturation stage. Each treatment consisted of nine replicate pots (three WT plants and three plants of each transgenic line, one plant per pot). Each ethylene stress treatment consisted of nine replicate trays (three detached functional leaves from the WT and each transgenic line). All experiments were replicated three times and randomized. The regeneration frequency of Km‐resistant shoots and the *NPTII*‐positive frequency of the explants were calculated as previously described 2. The data were recorded and analyzed statistically using SAS version 6.12 (SAS Institute, 1995, Cary, NC, USA). Significant differences among means were compared using Duncan's Multiple Range Test. All stress experiments were conducted in a greenhouse at 28 ± 2 °C/23 ± 2 °C (day/night) with a relative humidity of 50%‐70% under a 16/8‐h (light/dark) photoperiod and a photon flux density of 350 μmol·m^−2^·s^−1^.

## Results and Discussion

### Generation of transgenic ramie plants and transformation efficiency

**Figure 2:**
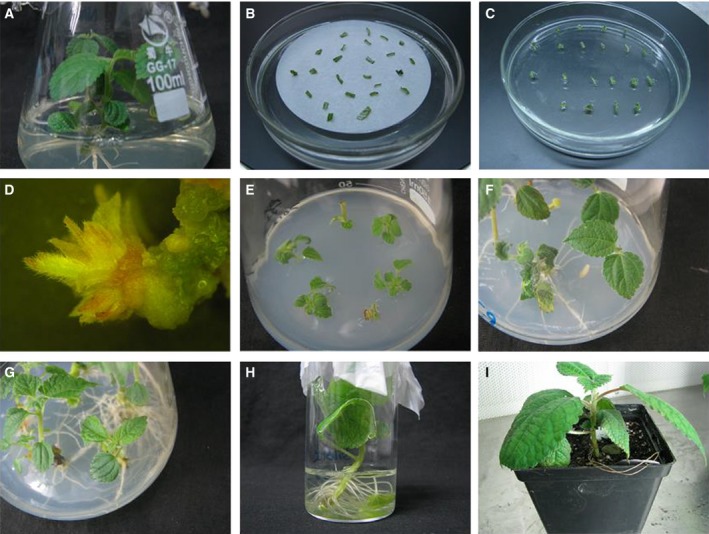
Generation of transgenic ramie plants. (A) 40‐day‐old plants grown on MS medium supplemented with 0.01 mg·L^−1^ NAA were used for leaf midrib explant preparation. The triangular flask is 6 cm in diameter and 10 cm in height. (B) Leaf midrib explants were infected with EHA105 harboring pSG529 and cultured on solid cocultivation medium (MS medium + 0.2 mg·L^−1^ TDZ + 0.04 mg·L^−1^ 2,4‐D + 50 mg·L^−1^ AS). The diameter of Petri dish is 9 cm. (C) After 2 days’ coculture, leaf midrib explants were incubated on selection medium (MS + 0.2 mg·L^−1^ TDZ + 0.04 mg·L^−1^ 2,4‐D + 40 mg·L^−1^ kanamycin+750 mg·L^−1^ cefotaxime). (D) Infected leaf midrib explants on selection medium produced Km‐resistant shoots after 3 weeks of selection culture. (E) Transgenic plants were cultured on elongation medium (MS medium + 250 mg·L^−1^ cefotaxime + 0.01 mg·L^−1^ NAA). (F) Transgenic ramie plants rooted. (G) Transgenic shoots propagated on MS medium supplemented with 0.01 mg·L^−1^ NAA. (H) Transgenic plants grown in Hoagland's solution. The cylindrical bottle is 4 cm in diameter and 10 cm in height. (I) Transgenic ramie plants were transplanted to small plastic pots in the greenhouse. The length, width, and height of the pots are 11, 11, and 14 cm, respectively.

**Figure 3:**
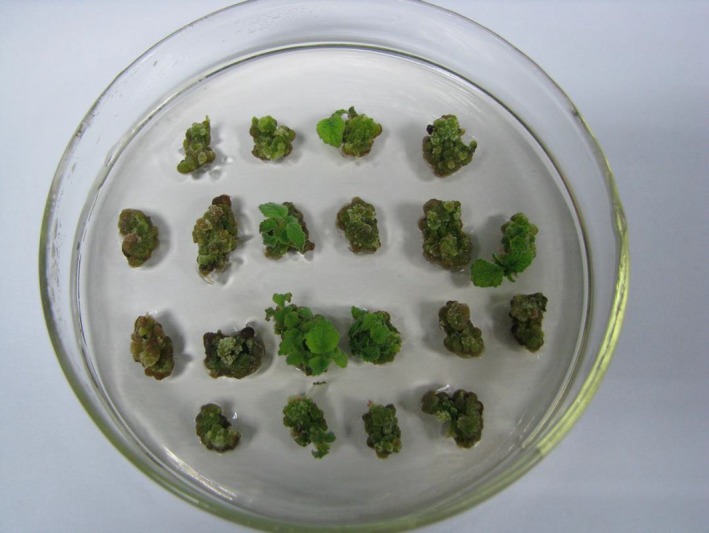
The regeneration of transgenic plants. The diameter of Petri dish is 9 cm.

The explant regeneration and transformation were shown in Fig. 2. Delaying senescence using the P_SAG12_‐*ipt* gene construct has also been reported in lettuce 23, *Petunia* flowers 24, *Arabidopsis* 34, gerbera 35, and creeping bentgrass 32. Regeneration and transformation were performed using midrib explants from 40‐day‐old *in vitro* shoots, an *Agrobacterium* concentration at OD_600_ of 0.6, 4‐min immersion in the bacterial solution, an acetosyringone concentration of 50 mg·L^−1^ in the cocultivation medium and a 2‐day cocultivation period. The efficiencies of regeneration and transformation were 28.29% and 25.56%, respectively. The regeneration of transgenic plants was shown in Fig. 3. In this study, the regeneration frequency of Km‐resistant shoots was higher than that previously reported 2.

### Molecular analysis of the transgenic plants

**Figure 4:**
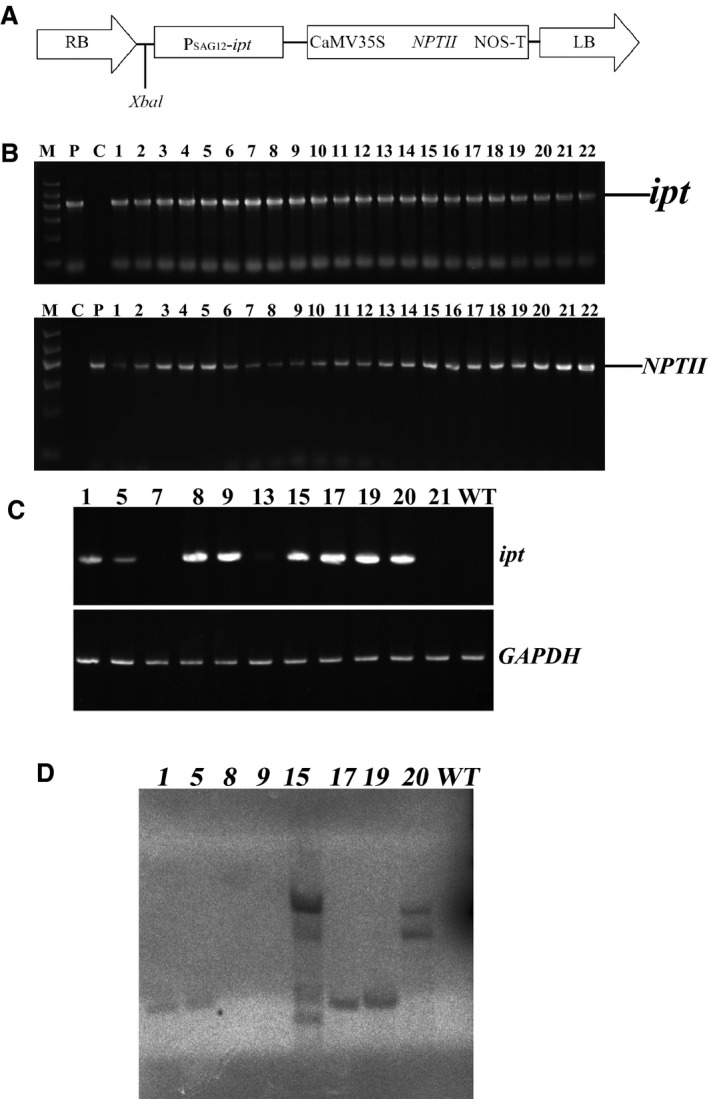
The vector construct and molecular characterization of transgenic ramie plants. (A) Schematic drawings of the T‐DNA region in the pSG529 plasmid vector. *RB*, T‐DNA right border; P_*SAG*__*12*_ *‐ipt*, the cytokinin biosynthesis gene *ipt*, derived from *Agrobacterium tumefaciens*, under the control of the *SAG12* promoter from *Arabidopsis*;* CaMV35S,* cauliflower mosaic virus; *NPTII*, neomycin phosphotransferase II gene; *NosT*, nopaline synthase terminator; *LB*, T‐DNA left border. *Xbal* was the restriction enzyme site. (B) PCR analysis of the transgenic plants using primers specific to the *ipt* and *NPTII* genes. M, molecular size marker [the first lane is the DNA marker (TIANGEN Biotech Co. Ltd., Beijing, China)]. The fragment lengths of the DNA marker were 100 bp, 300 bp, 500 bp, 700 bp, 900 bp, and 1200 bp. P, positive control (plasmid DNA); C, control (untransformed plant). Lanes 1–22 were transgenic lines 1–22, respectively. Arrows point to the expected size bands. (C) Total RNA was isolated from WT and transgenic lines 1, 5, 7, 8, 9, 13, 15, 17, 19, 20, and 21. RT‐PCR was performed with *ipt*‐specific or *GAPDH*‐specific primers. The *GAPDH* gene was used as an internal control. (D) In the Southern blot analysis of the transgenic plants, the number of bands reflects the number of transgene insertions. Lane 9 was a negative control. Lanes 1–8 are transgenic lines 1, 5, 8, 9, 15, 17, 19, and 20, respectively.

The binary vector pSG529 harboring the Ck biosynthesis gene *ipt*, derived from *A. tumefaciens*, under the control of the SAG12 promoter from *Arabidopsis* was shown in Fig. 4A. Vegetative propagation is the main propagation method in practical ramie production and the materials here did not produce seeds; all plants used in this study were thus T0 plants 33. Twenty‐two putatively transgenic T0 ramie plants were regenerated and confirmed by PCR (Fig. 4B). Eleven of them (lines 1, 5, 7, 8, 9, 13, 15, 17, 19, 20, and 21) were randomly selected for RT‐PCR (Fig. 4C), among which lines 1, 5, 8, 9, 15, 17, 19, and 20 had the highest *ipt* transcript levels and were chosen for further analysis. Copy number analysis showed that the six T0 lines (lines 1, 5, 15, 17, 19, and 20) had different numbers of inserts (Fig. 4D).

### Analysis of chlorophyll content and photosynthesis in fresh leaves at the fiber maturation stage

**Figure 5:**
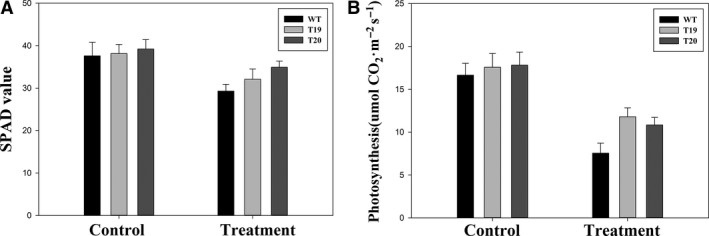
Transgenic plants expressing *ipt* had higher SPAD values and photosynthesis rates than WT plants. The SPAD values (A) and photosynthesis rates (B) of these seedlings were measured accordingly with triplicates. Asterisks in (A) and (B) indicated significant differences (*P* < 0.05) between the transgenic lines and WT plants.

The leaf relative chlorophyll content (SPAD values) and photosynthesis rates of randomly selected transgenic and WT plants were measured at the beginning (control) and end (treatment) of the fiber maturation stage. There were no differences in SPAD values or photosynthesis rates among the randomly selected transgenic and WT plants at the beginning of the fiber maturation stage. As expected, leaf senescence and leaf abscission at the fiber maturation stage had an adverse effect on SPAD values and photosynthesis rates (Fig. 5A,B; Treatment). However, the reductions in SPAD values and photosynthesis rates of the transgenic plants were less severe than those observed in the WT plants (Fig. 5A,B; Treatment). Leaf senescence is a type of programmed cell death (PCD) characterized by loss of chlorophyll, lipids, total protein, and RNA 36. The potential for improvement of crop characteristics, particularly plant productivity and postharvest storage, has prompted extensive physiological, molecular, and genetic analyses of leaf senescence 37. Endogenous factors, including leaf age and reproductive development, trigger senescence 36. Leaf senescence at the fiber maturation stage is a biological process. In the WT, leaf senescence was initiated at the fiber maturation stage in naturally grown plants, which turned yellow (low SPAD values), whereas senescing leaves from the P_SAG12_‐*ipt* transgenic plants remained dark green (high SPAD values). As leaves are the main photosynthetic organs, the expression of *ipt* in leaves resulted in maintenance of chlorophyll content and higher photosynthesis rates compared with WT plants in this study.

### Ethephon‐induced leaf senescence analysis

**Figure 6:**
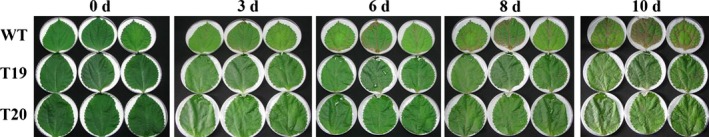
Effect of ethephon‐induced leaf senescence. Plants were subjected to 1200 ppm ethephon treatments for 10 days and photographed. The diameter of Petri dish is 15 cm. See ‘Materials and methods’ for further details.

Ethylene is known to promote the leaf senescence process 29. To confirm that leaf senescence was delayed in *ipt*‐overexpressing plants, an ethephon‐induced leaf senescence test was conducted using the above two *ipt*‐overexpressing plants (T19 and T20) and WT plants. Detached leaves of the transgenic and WT plants were incubated in the presence of 1200 ppm ethylene for 10 days in water (mock treatment) and photographed. After ethephon treatment for 3 days, WT leaves showed visible leaf yellowing, while the transgenic lines displayed delayed leaf senescence (Fig. 6). Ethephon treatment for 6 days had a clear effect on the WT plants, whereas transgenic lines 19 and 20 remained green (Fig. 6). Ethephon treatment for 10 days had a severe effect on the WT plants, which were completely wilted, whereas transgenic lines 19 and 20 showed only slight yellowing (Fig. 6). Therefore, our results indicated that ethephon‐induced leaf senescence was delayed in the transgenic lines compared with WT plants. A reduction in sensitivity to ethylene has also been reported in P_SAG12_‐*ipt* transgenic petunia plants, where increased levels of Cks and *ipt* mRNA abundance were detected a few hours after ethylene treatment 24.

### Analysis of biomass traits and phenotype in transgenic and WT plants

**Figure 7:**
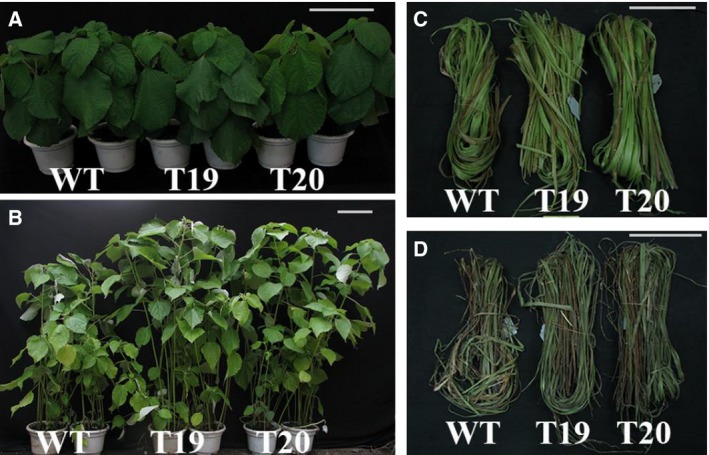
The phenotype of transgenic and WT plants. Plants at (A) the beginning (control) and (B) the end (treatment) of the fiber maturation stage. Subsequently, their stem basts were peeled (C) and dried (D). Bars are 10 cm.

**Figure 8:**
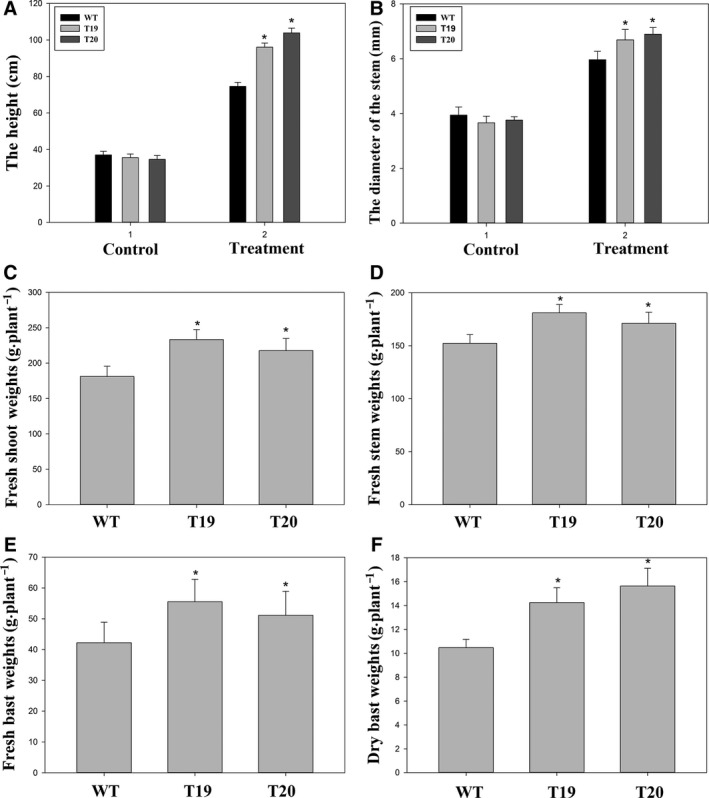
The biomass traits of WT and transgenic plants. Asterisks indicated significant differences (*P* < 0.05) between the transgenic lines and WT plants.

To confirm that biomass was increased in the *ipt*‐overexpressing plants, the phenotype and biomass traits including height, stem diameter, fresh shoot weight, fresh stem weight, fresh bast weight, and dry bast weight were measured. At the beginning of the fiber maturation stage, there were no significant differences among the WT and transgenic plants in phenotype (Fig. 7A), height, or stem diameter (Fig. 8, control). However, there were significant differences between the WT and transgenic plants in height, stem diameter, fresh weight, fresh stem weight, fresh bast weight, and dry bast weight (Fig. 8, treatment) at the end of the fiber maturation stage. At the fiber maturation stage, significant phenotypic differences (Fig. 7B) between transgenic lines 19 and 20 were observed. Our data showed that transformation of ramie with the P_SAG12_‐*ipt* gene delayed leaf senescence and enhanced biomass. Roses and stay‐green lines have been obtained with similar features to those described in the present paper for ramie 29. Because of the worldwide economic importance of ramie, the decreased senescence observed in transgenic P_SAG12_‐*ipt* ramie has commercial value. The transgenic plants described here provide a range of stay‐green phenotypes to facilitate studies of the impact of leaf senescence manipulation on yield under field conditions.

## Conclusions

In this study, the transformation efficiency was 25.56%, which was higher than that reported previously 2. The transgenic plants had higher photosynthesis rates and SPAD values than wild‐type plants at the fiber maturation stage. The transgenic plants also had enhanced resistance to exogenous ethylene compared with the wild‐type plants at the fiber maturation stage. Overall, this study suggests that *ipt* gene overexpression delayed leaf senescence and enhanced biomass characteristics including height, stem diameter, fresh weight, fresh stem weight, fresh bast weight, and dry bast weight in transgenic plants compared with WT plants, and has the potential to improve ramie fiber yield.

## Author contributions

XA, JYZ, and YWL carried out the sample preparation, analyzed results, and drafted the manuscript. XA and BW conceived and designed the experiments. LJL and DXP contributed reagents and materials. All authors read and approved the final manuscript.
